# Supplementary material for: Syngas Production Improvement from CO2RR Using Cu-Sn Electrodeposited Catalysts
Source: Materials (Basel). 2024 Dec 30;18(1):105. doi: 10.3390/ma18010105 (PMC11722079; doi:10.3390/ma18010105)
Supplement: Supplementary file 1 [file materials-18-00105-s001.zip › materials-3357284-supplementary.pdf]

Supporting information for

Syngas Production Improvement from CO2RR Using Cu-Sn Electrodeposited Catalysts

Daniel Herranz [1], Santiago Bernedo Biriucov [1], Antonio Arranz [2], Juan Ramón Avilés Moreno [1,*] and Pilar Ocón [1]

[1] Departamento de Química Física Aplicada, Universidad Autónoma de Madrid (UAM), C/Francisco Tomás y Valiente 7, 28049 Madrid, Spain; daniel.herranz@uam.es (D.H.); santiago.bernedo@estudiante.uam.es (S.B.B.); pilar.ocon@uam.es (P.O.)

[2] Departamento de Física Aplicada, Universidad Autónoma de Madrid (UAM), C/Francisco Tomás y Valiente 7, 28049 Madrid, Spain; antonio.arranz@uam.es

* Correspondence: juan.aviles@uam.es

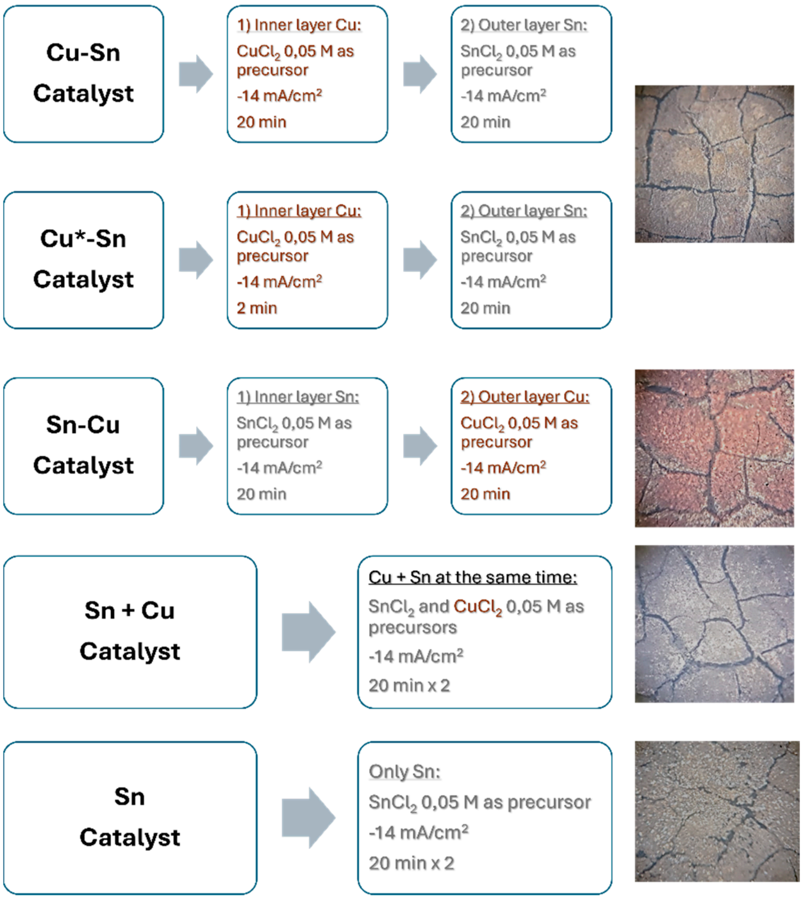

Figure S1. Electrodeposition conditions scheme of the five catalysts studied in this work.

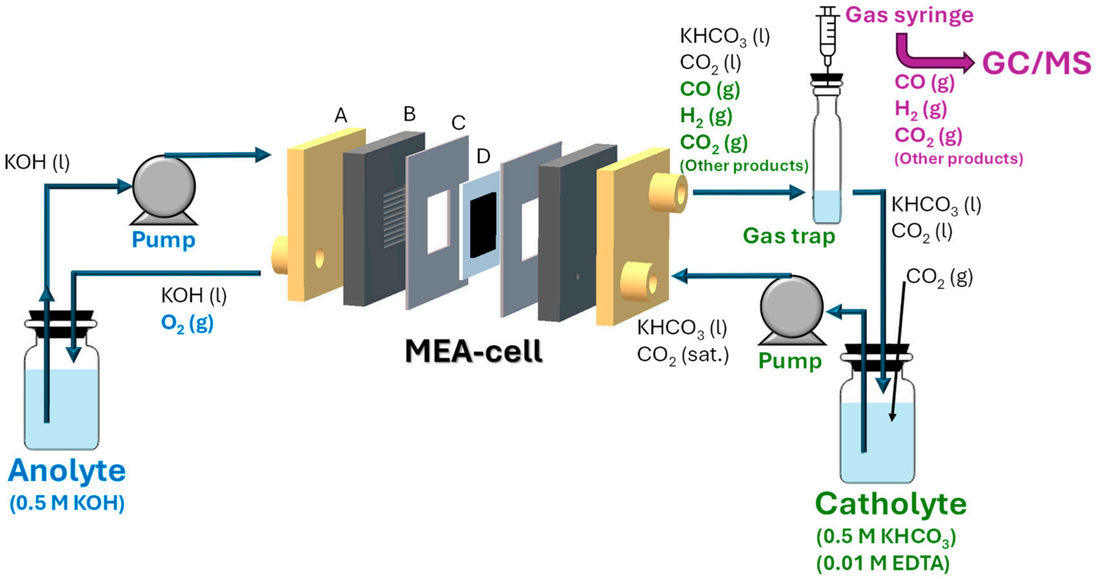

Figure S2. Experimental setup for CO2RR experiments. Components of the MEA-cell: A (current collector), B (bipolar plate with flow channels), C (gasket), D (MEA). The membrane-electrode assembly (MEA) is composed of the anode catalyst (Ni foam), the anion exchange membrane and the cathode catalyst.

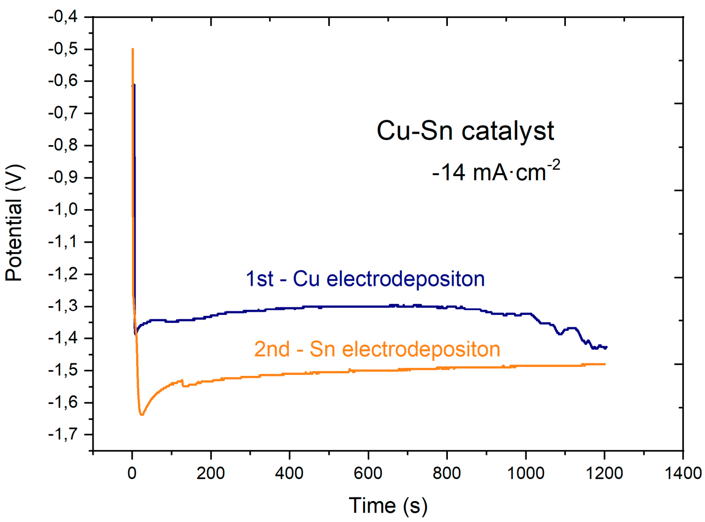

Figure S3. Example of CPs obtained during electrodepositions of metals on the carbon cloth. Potential values are vs. Ag/AgCl (KCl sat.) reference electrode.

Table S1. Comparison of Cu-Sn, Sn-Cu and Sn+Cu catalysts performance at a constant flow and different currents densities.

Catalysts	Flow ($mL\cdot min^{-1}$)	Current ($mA\cdot cm^{-2}$)	Voltage (V)	$\%CO_2$ outlet	FE to products			
					$\%H_2$	$\%CH_4$	$\%C_2H_4$	%CO
Cu-Sn	80	-25	-2.02	36	66	0	0	34
	80	-50	-2.23	43	89	0	0	11
	80	-100	-2.58	35	96	0	0	4
	80	-200	-2.8	28	99	0	0	1
Sn-Cu	80	-25	-1.96	53	69	0	0	31
	80	-50	-2.11	49	71	0	0	29
	80	-100	-2.45	37	76	0	0	24
	80	-200	-3,00	35	92	<	0	8
Cu+Sn	80	-25	-1.95	54	93	0	0	7
	80	-50	-2.19	59	77	0	0	23
	80	-100	-2.59	46	71	<1	<1	29
	80	-200	-3.5	39	81	4	<1	14

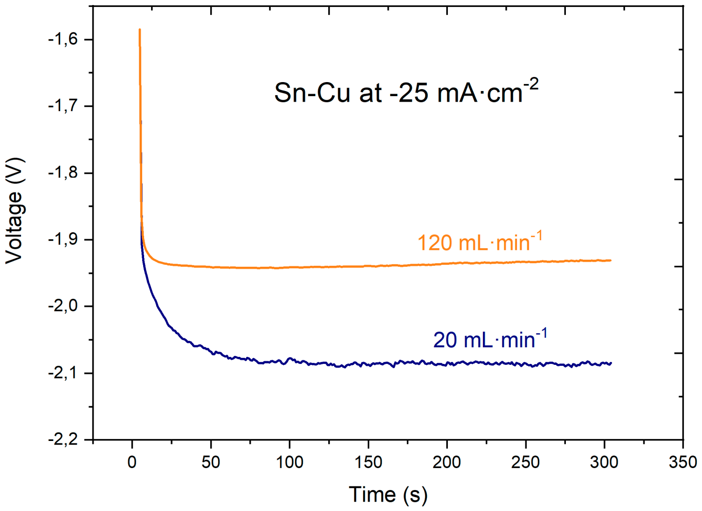

Figure S4. Example of CPs obtained during CO2RR with Sn-Cu catalyst at two different electrolyte flow rates with current density of -25 mA·cm-2.

Table S2. Comparison of Cu-Sn, Sn-Cu and Sn+Cu catalysts performance at a constant current density of -25 mA·cm^{-2} and different electrolyte flow rates.

Catalysts	Flow (mL·min^{-1})	Current (mA·cm^{-2})	Voltage (V)	%CO_2 outlet	FE to products			
					%H_2	%CH_4	%C_2H_4	%CO
Cu-Sn	20	-25	-2.18	40	83	0	0	17
	40	-25	-2.08	46	72	0	0	28
	80	-25	-2.08	33	66	0	0	34
	120	-25	-1.98	44	54	0	0	46
Sn-Cu	20	-25	-2.08	41	68	0	0	32
	40	-25	-2.01	47	66	0	0	34
	80	-25	-1.96	53	69	0	0	31
	120	-25	-1.93	56	73	0	0	27
Cu+Sn	20	-25	-2.06	33	89	0	0	11
	40	-25	-1.96	46	91	0	0	9
	80	-25	-1.95	54	93	0	0	7
	120	-25	-1.93	59	93	0	0	7

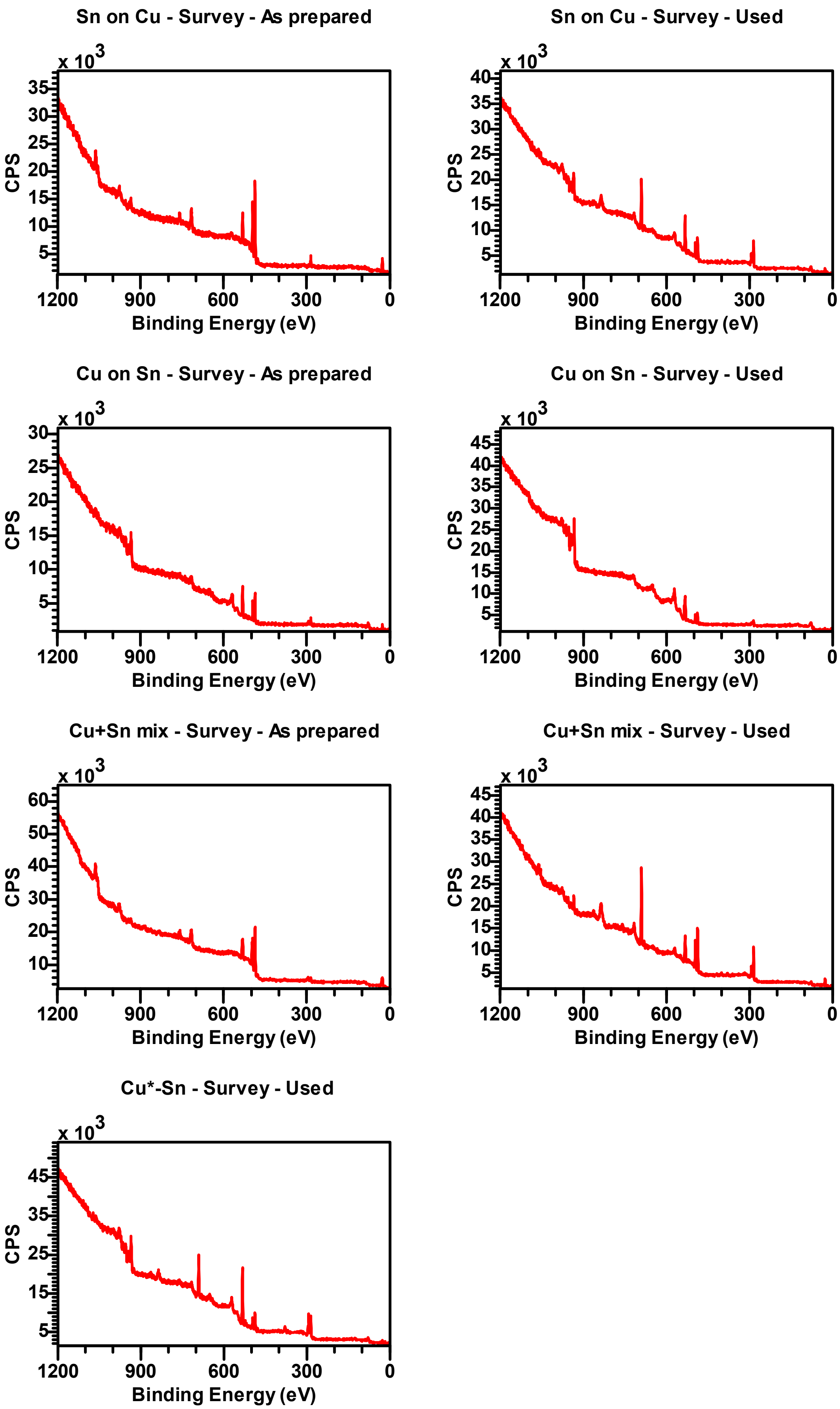

Figure S5. Survey XPS spectra.

Table S3. Comparison of Cu*-Sn and Sn catalysts performance with different electrolyte flow rates and current densities.

Catalysts	Flow ($mL\cdot min^{-1}$)	Current ($mA\cdot cm^{-2}$)	Voltage (V)	$\%CO_2$ outlet	FE to products			
					$\%H_2$	$\%CH_4$	$\%C_2H_4$	%CO
Cu*-Sn	80	-25	-2.05	48	38	0	2	60
	80	-50	-2.30	28	82	<1	5	13
	80	-100	-2.50	28	95	<1	2	3
	80	-200	-2.80	25	98	<1	1	1
Sn	80	-25	-1.99	49	88	0	0	12
	80	-50	-2.33	44	93	0	0	7
	80	-100	-3.00	39	97	0	0	3
	80	-200	-3.5	32	99	0	0	1
Cu*-Sn	20	-25	-2.2	38	78	0	1	21
	40	-25	-2.11	43	54	0	2	44
	80	-25	-2.05	48	38	0	2	60
	120	-25	-2.00	49	37	0	1	62
Sn	20	-25	-2.14	44	95	0	0	5
	40	-25	-2.1	40	90	0	0	10
	80	-25	-1.99	49	88	0	0	12
	120	-25	-1.96	53	97	0	0	3

Cyclic voltammetry (CV) at various scan rates (5-100 $mV\cdot s^{-1}$) in a non-faradaic region of potentials was used to measure the electrode double layer capacitance (C_{DL}). The range of potential was different depending on the catalyst: (-0.6V to -0.2V) for Cu*-Sn and Cu-Sn, (-0.2 to +0.4V) for Sn and Cu+Sn and (-0.45V to -0.1V) for Sn-Cu and (-0.7V to +0.4V) for Cu. The current density values were taken at the middle of each CV. The C_{DL} was determined as slope of Equation S1.

$$\Delta J_{Capacitive} = (J_{Anodic} - J_{Cathodic}) = C_{DL} \cdot SR + a_{Intercept} \qquad \text{(Eq. S1)}$$

were $\Delta J_{Capacitive}$ is the variation of current density, J_{Anodic} and $J_{Cathodic}$ are the anodic and cathodic current densities (all of them respect to the geometrical surface area), C_{DL} is the double layer capacitance, SR is the scan rate and $a_{Intercept}$ is the intercept value with the Y axis in the representation. The difference between the anodic and cathodic current densities is linearly dependent from the scan rate at a fixed potential.

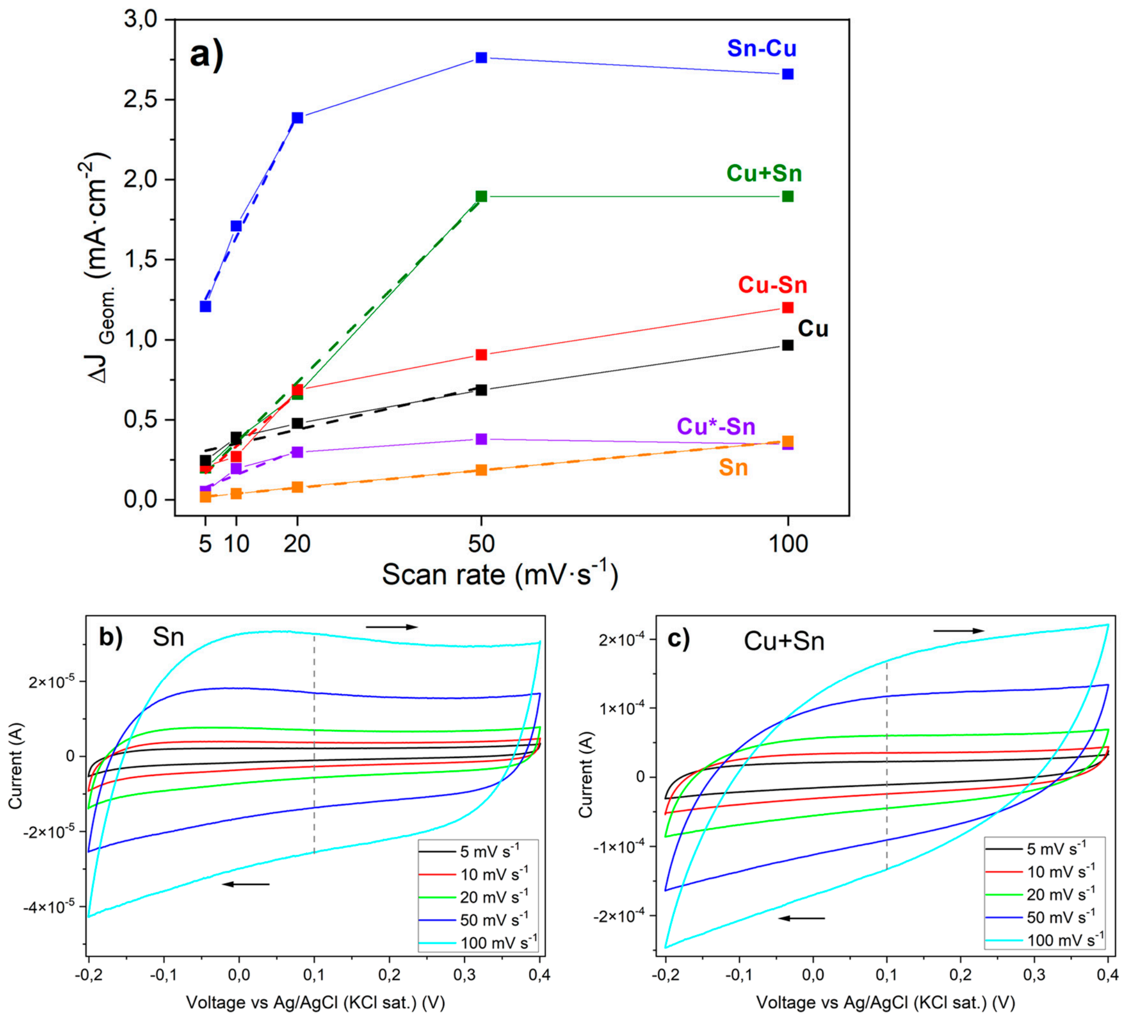

Figure S6. a) Capacitive current density (variation of geometrical current density) at a fixed potential vs Scan rate. Cyclic voltammograms at various scan rates in a non-faradaic region of potentials for: b) Sn and c) Cu+Sn.

If Sn is used as a reference to normalize the C_{DL} values of the rest of the catalysts, the values included in Table S4 give the following trend:

Sn < Cu < Cu*Sn < Cu-Sn < Cu+Sn < Sn-Cu

Table S4. Double layer capacitance (C_{DL}) values of the prepared catalysts and normalization respect to the lowest results.

Catalyst	C_{DL} ($F·cm^{-2}$)	R^2 linear fit	Normalization
Sn	0,0036	1,000	1
Cu*-Sn	0,0155	0,922	4
Cu-Sn	0,0330	0,954	9
Cu+Sn	0,0380	0,995	11
Sn-Cu	0,0770	0,988	21
Cu	0,0088	0,931	2

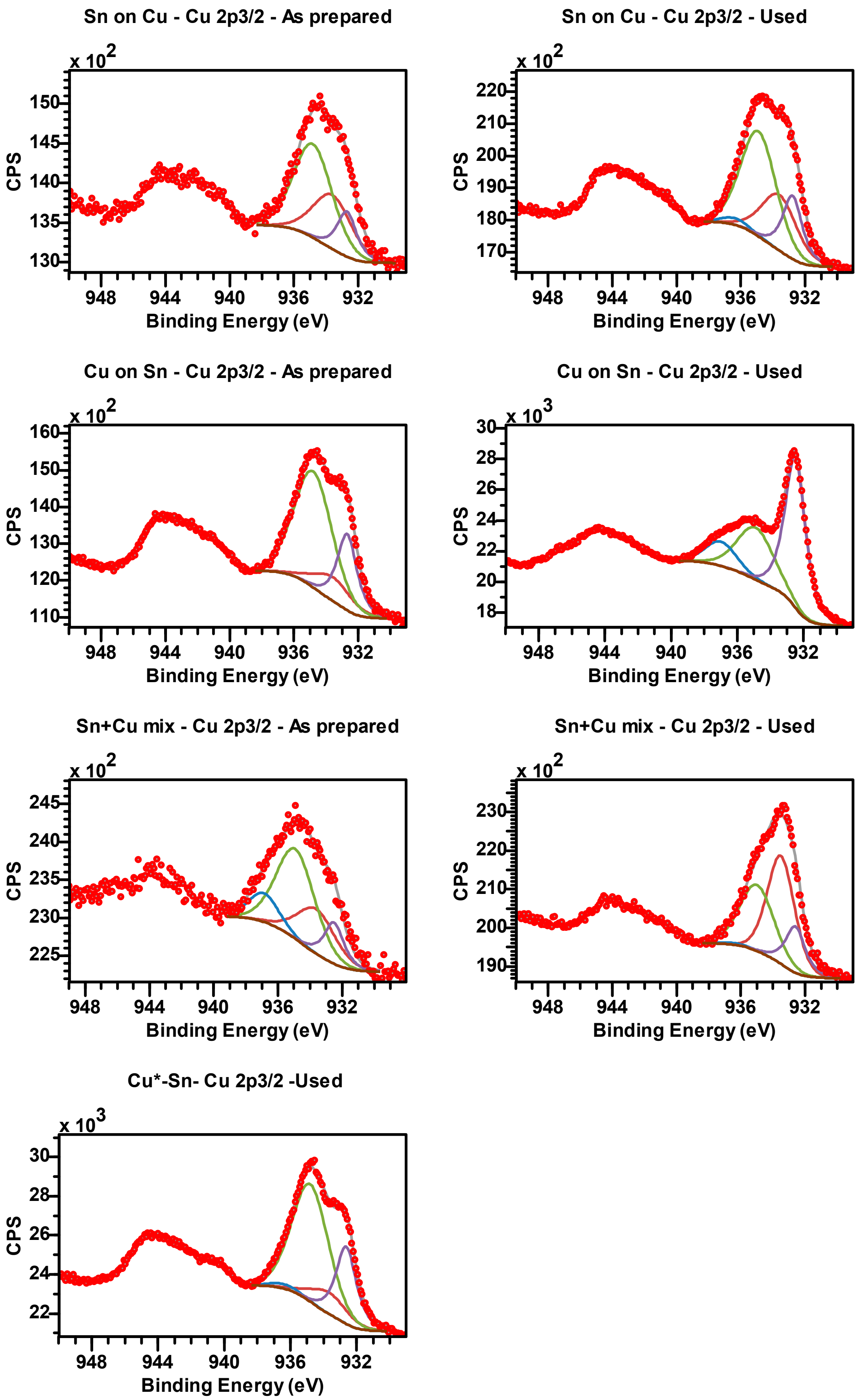

Figure S7. XPS deconvolution of Cu $2p_{3/2}$. The overlapping Cu^0 and Cu^+ species are shown in violet. Cu^{2+} species were ascribed to CuO (brown), $Cu(OH)_2$ (green) and CuF_2 (blue, F coming from Nafion® in the microporous carbon layer of the carbon cloths).

The Cu $2p_{3/2}$ core level spectra shown in Figure S5 have a complex structure. The secondary feature at higher binding energies is the well-known shake-up satellite characteristic of Cu^{2+} species [46], whereas the main peak at lower binding energies is the Cu $2p_{3/2}$ core level peak itself. To get additional insight into the different Cu chemical species, a deconvolution procedure has been carried out for the Cu $2p_{3/2}$ main peak. Four synthetic peaks at 932.6 ± 0.1, 933.6 ± 0.1, 934.9 ± 0.1 and 936.4 eV, with a full width at half maximum (fwhm) of 1.45, 2.7 ±0.1, 2.7 ± 0.1 and 2.1 ± 0.1 eV, respectively, have been used to account for overlapping Cu_2O+Cu^0 (violet), CuO (brown), $Cu(OH)_2$ (green) and CuF_2 (blue), respectively. It should be pointed out that a slight variation of the fwhm of the peaks associated with Cu^{2+} chemical species has been necessary to properly fit spectra of Figure S5 (new Figure Sx).

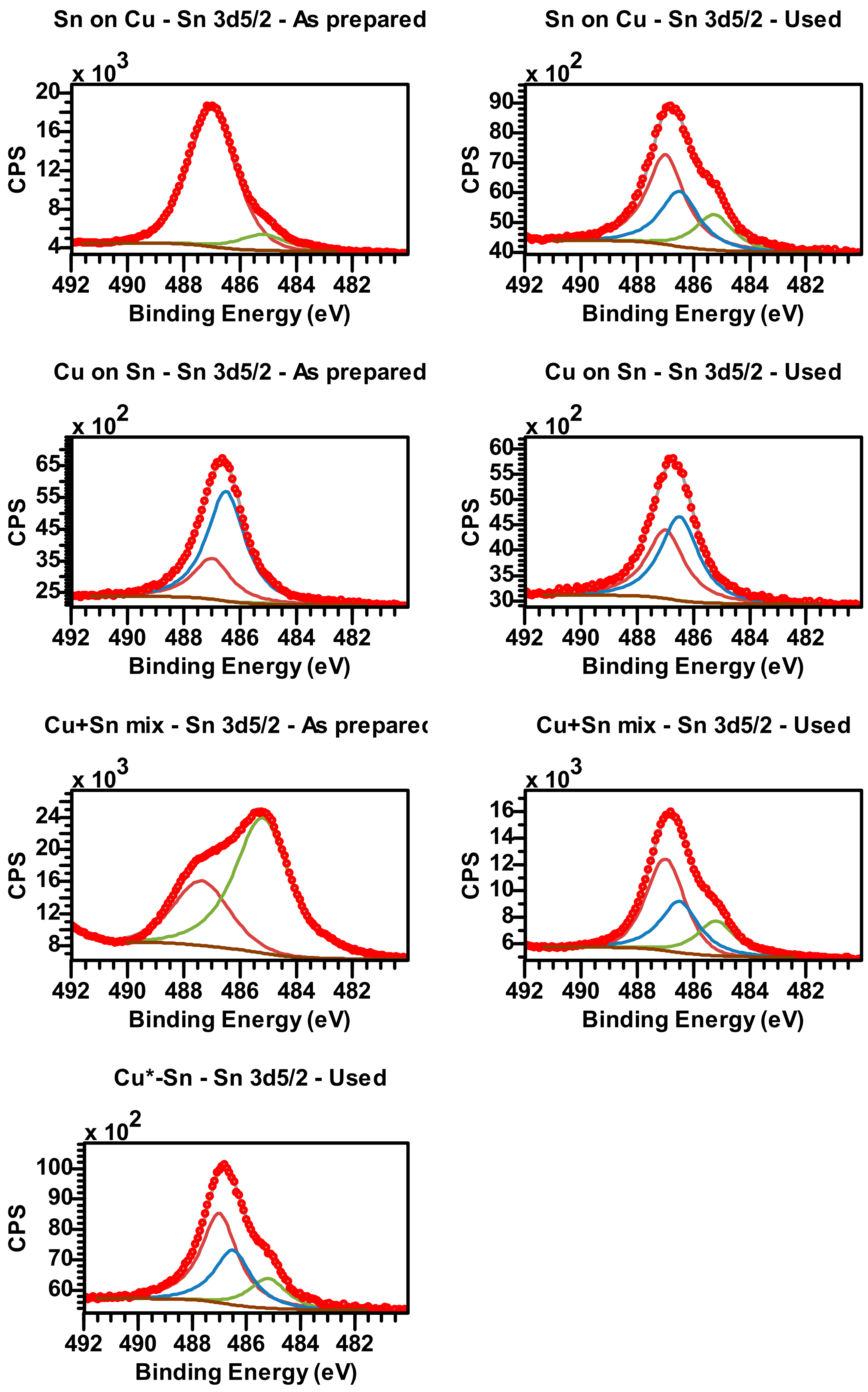

Figure S8. XPS deconvolution of Sn 3d5/2. Species are Sn^0 (green), Sn^{2+} from SnO (blue) and Sn^{4+} from SnO_2 (brown).

Sn $3d_{5/2}$ core level spectra are shown in Figure S6. To get additional insight, a deconvolution procedure has been carried out for this core level. Three synthetic peaks at 485.2, 486.5 and 487.0 eV, with the same fwhm of 1.6 eV (except for the "Cu+Sn mix" sample, in which a greater fwhm has been used due to charging effects) have been used to account for Sn^0 (green), SnO (blue) and SnO_2 (brown) chemical species, respectively.

Table S5. Comparison of Cu*-Sn catalyst stability performance with different electrolyte flow rates and current densities.

Catalyst use	Flow ($mL·min^{-1}$)	Current ($mA·cm^{-2}$)	Voltage (V)	$\%CO_2$ outlet	FE to products			
					$\%H_2$	$\%CH_4$	$\%C_2H_4$	%CO
1st	80	-25	-2.05	48	38	0	2	60
	80	-50	-2.30	28	82	<1	5	13
	80	-100	-2.50	28	95	<1	2	3
	80	-200	-2.80	25	98	<1	1	1
2nd	80	-25	-1.77	44	59	0	<1	41
	80	-50	-2.21	37	78	0	8	14
	80	-100	-2.51	27	92	<1	3	4
	80	-200	-2.74	44	98	<1	1	1
3th	80	-25	-1.96	40	72	0	2	26
	80	-50	-2.19	37	79	0	6	15
	80	-100	-2.39	31	93	<1	3	3
	80	-200	-2.55	12	99	<1	<1	<1
4th	80	-25	-2.04	53	75	0	1	24
	80	-50	-1.99	44	93	0	0	7
1st	20	-25	-2.20	38	78	0	1	21
	40	-25	-2.11	43	54	0	2	44
	80	-25	-2.05	48	38	0	2	60
	120	-25	-2.00	49	37	0	1	62
2nd	80	-25	-1.77	44	59	0	<1	41
	120	-25	-1.90	47	66	0	2	32
3th	20	-25	-2.16	22	97	0	2	2
	40	-25	-2.06	32	84	0	4	13
	80	-25	-1.96	40	72	0	2	26
	120	-25	-1.92	43	71	0	1	28
4th	20	-25	-2.24	30	96	0	1	3
	80	-25	-2.02	53	75	0	1	24
